# Mesenchymal Stem/Progenitor Cells and Their Derivates in Tissue Regeneration—Part II

**DOI:** 10.3390/ijms25094937

**Published:** 2024-04-30

**Authors:** Aleksandra Klimczak

**Affiliations:** Laboratory of Biology of Stem and Neoplastic Cells, Hirszfeld Institute of Immunology and Experimental Therapy, Polish Academy of Sciences, R. Weigla 12, 53-114 Wroclaw, Poland; aleksandra.klimczak@hirszfeld.pl

During the last three decades, mesenchymal stem/stromal cells (MSCs) were extensively studied, and are mainly considered within the setting of their regenerative and immunomodulatory properties in tissue regeneration. Cells bearing the characteristics of MSCs can be isolated from a variety of tissues of the adult organism (including bone marrow, adipose tissues, skin, skeletal muscles, synovial membranes, tendons, bones, peridental tissues, liver, lungs, and others), and from perinatal tissues (such as cord blood or Wharton jelly) [1]. In the steady-state conditions, MSCs and tissue-specific progenitor cells, residing in almost all adult organs, participate in tissue regeneration to maintain homeostasis. However, despite common MSCs characteristics, our earlier studies revealed differences between MSCs derived from various tissues in terms on their surface markers and their multipotential ability to differentiate into other lineages [2]. It is now widely accepted that not only intact MSCs, but also their derivates, especially MSC-secretome and MSC-derived extracellular vesicles (MSC-EVs), hold immunomodulatory, anti-inflammatory, antiapoptotic, and proangiogenic properties, due to secretion of a wide range of bioactive factors; thus, they may modulate local environment, leading to tissue regeneration [3]. Many studies showed that the regenerative effects are attributed to the cooperation of several types of MSC-derived secretomes, including extracellular vesicles (EVs) and soluble factors [4]. Extracellular vesicles are very small nanoparticles commonly classified for exosomes (Exo), microvesicles (MVs), and apoptotic bodies, based on their size and intracellular origin, according to the updated Minimal Information for Studies of Extracellular Vesicles 2018 (MISEV2018) guidelines [5]. At the early stage of research, potential cell therapies using MSCs were considered as a promising tool for clinical application in the treatment of many diseases. However, for cell-based therapy using MSCs to be considered effective and safe, we need detailed characteristics of cells or their derivatives that will constitute a medicinal product. 

All the papers that contributed to this Special Issue, presenting the latest data and opinions, can add new insights for elucidating the pro-regenerative potential of MSCs and their secretome in tissue regeneration.

Large bone defects following traumas or tumor resections after orthopedic or oral–maxillofacial surgeries are very difficult pathological conditions, and require extensive bone regeneration. A promising alternative to treat clinically challenging bone defects becomes an interdisciplinary approach via bone tissue engineering. The potential beneficial effects of different bone tissue engineering components, including MSCs, biomaterial scaffolds, and growth factors involved in osteogeneic processes, could provide a new therapeutic opportunity for critical size bone defects [6]. Contribution #1 of this Special Issue introduce new findings on the role of MSCs supporting a scaffold in bone regeneration. This study explored the osteogenic potential of human dental pulp stem cells (hDPSCs) grown on a hydrolytically modified poly(L-lactide-co-caprolactone) (PLCL) electrospun scaffold and a non-woven hyaluronic acid (HYAFF-11™) mesh. The results revealed that PLCL scaffolds provide a better environment that supports hDPSC adhesion and osteogenic differentiation than HYAFF-11™ meshes, as confirmed by SEM analysis. The high mRNA expression of early (*Runx2*, *Coll-I*, *Osx*) and late (*Opn*, *Bsp*) osteogenic genes and mineral deposits observed after hDPSC osteogenesis on a PLCL mat indicated its better impact on hDPSCs’ osteogenic potential than that of HYAFF-11™. This study showed that the hDPSC/PLCL construct might be further considered as an innovative approach for bone defect repairs.

The regenerative activity of MSCs is related to the composition of the MSCs secretory profile, which also depends on the tissue source from which they were isolated. Contribution #2 introduces a study of the changes in the MSCs functional activity under exposure to X-ray radiation at low and medium doses, which is particularly important, because MSCs play the role of a regenerative reserve in the human body and have the ability to self-renew and the potential to differentiate into desired cells, thus maintaining tissue homeostasis.

In addition to the pro-regenerative potential of intact MSCs, the covered topics of this Special Issue include studies on bioactive factors and extracellular vesicles released by MSCs for cell-free therapy in tissue regeneration. Mesenchymal stem/stromal cell-conditioned medium (CM) provides a potential opportunity for the treatment of skin diseases, including wound healing [7,8]. As previously reported, a comparable therapeutic effect to MSCs can also be achieved through their secretome, which contains bioactive factors and cytokines secreted by parental cells. Our study on cell-free therapy in chronic wounds showed that a conditioned medium from MSCs of an adipose tissue-origin revealed the presence of variety of bioactive factors (e.g., IL-8, VEGF, IGF-1, MCP-1, FGF, and others), with anti-inflammatory properties and trophic factors promoting dermal fibroblasts proliferation, and proangiogenic growth factors that modulate the ischemic microenvironment and augment angiogenesis [7].

Another difficult-to-treat chronic disease is tendon damage, characterized by the accumulation of inflammatory cells and the induction of a local inflammatory response caused by the production of inflammatory cytokines, including TNF and IL-1. Persistent inflammation and a lack of effective treatment options play key roles in the progression of the disease, and lead to the transition to a chronic disease. The immunomodulatory properties of the MSC secretome are considered as a potential option for cell-free therapy for the treatment of tendinopathy by modifying the inflammatory microenvironment [9]. Contribution #3 of this Special Issue presents a very interesting study on the therapeutic effect of a conditioned medium (CM) obtained from a BM-MSC culture, its EV fraction, and its soluble protein fraction (PF, CM without EVs) on inflamed tenocytes of horse origin. This research has been carried out in equine cells, as horses suffer from naturally occurring tendinopathy analogous to humans, and represent a well-established animal model recommended by the U.S. Food and Drug Administration (FDA) and the European Medicines Agency (EMA). Studies showed that CM contained the highest concentrations of proteins and particles, relative to PF and EVs, respectively. Inflammation significantly altered the gene expression in tenocytes. Treatment with CM revealed its immunomodulatory capacity, which led to the most significant differential gene expression, associated with both up- and down-regulation of genes responsible for inflammation and tissue regeneration. EV treatment also produced a therapeutic effect, but it was less effective compared to CM. This study demonstrated that treatment with a complete CM has a greater therapeutic potential than EVs or PF fractions alone, suggesting a possible synergistic effect of different secretome fractions in the treatment of tendinopathy. Recent studies on the mechanisms by which MSCs exert their therapeutic effects on damaged tenocytes revealed that MSCs transfer mitochondria to injured tenocytes, thus decreased apoptosis, promoted proliferation, and restored mitochondrial function in injured tenocytes [10].

The functions of the MSCs are regulated by different bioactive factors, inflammatory mediators, extracellular microenvironments, cell transduction signals, and the cell metabolism. The key organelles responsible for cell energy metabolism are mitochondria, as they are the primary site of oxidation of carbohydrates, fats, and adenosine triphosphate production (ATP) production. The mitochondria play a major role in regulation of MSCs’ self-renewal, multipotential differentiation, ageing, and apoptosis, thus are crucial to maintaining homeostasis and regulating the fate of the MSCs. Understanding the basis of the crosstalk between mitochondria and cell fate is of critical significance in terms of potential applications of MSCs in regenerative medicine [11,12]. Studies on MSC secretome revealed that MSCs can secrete not only EVs, but also mitochondria, allowing for their transport to cells from the microenvironment. In the study constituting Contribution #4, two fractions of MSC-derived EVs differing in size (about 250 nm called ectosomes and about 100 nm called exosomes) were studied. Detailed analysis of the structural and functional characteristics revealed components from all mitochondria compartments, including house-keeping mitochondria proteins and DNA as well as energy-related proteins such as membrane-localized proteins of complexes I, IV, and V, and soluble proteins involved in the Krebs cycle.

MSCs secretome or conditioned medium is a combination of bioactive factors in cell culture growth medium, and the starting point of several derived products. MSC-CM and its derivatives could be applied after injuries, and could mediate most of the beneficial regenerative effects of MSCs without the possible side effects of using the MSCs themselves. However, before the clinical application of these promising bio-pharmaceuticals, several issues, such as manufacturing protocols and quality control, must be addressed. MSC secretome can be considered a medical product of cell-free therapy, as it is proposed as an alternative for the treatment of various diseases that do not respond to conventional therapies, by exerting immunomodulatory or metabolic effects [13]. However, prior to clinical application, validation of MSC secretome production according to the criteria proposed by the FDA and EMA is necessary. This issue is discussed in the review paper constituting Contribution #5 of this Special Issue. Contribution #6 underlines the influence of the procedure for conditioned medium production, and indicates many factors (e.g., the tissue source of MSCs, cell expansion, cell passage number and confluence, conditioning period, cell culture medium, etc.) that may influence the quality of the secretome-derived product.

Recently, there has been much research on the intra-articular administration of MSCs for the management of osteoarthritis of the knee joint as an alternative treatment method to total knee arthroplasty [14,15]. The review article constituting Contribution #7 introduced the latest data on the molecular mechanisms of the action of MSCs and their potential therapeutic benefits in clinical applications, including in a randomized placebo-controlled trial, in patients suffering with painful knee osteoarthritis. The efficacy of intra-articular MSC delivery for knee OA remains debatable, and a standardized protocol is needed, including: cell selection, characterization according to the International Society for Cellular Therapy recommendation (phenotypic analysis and multipotent differentiation potential) [16], culture or expansion techniques dosages, and a rehabilitation program after treatment with intra-articular MSC injections in knee OA. 

In summary, MSCs, or their secretome, can be used for therapy in several clinical indications, but MSCs may exhibit different functional properties, depending on the way in which they are produced, the type of MSCs product (intact cells or their secretome), and the route of administration (local, systemic). The complexity of MSC production to achieve medicinal products need standardization and cooperation between the research and clinician community across the world.
